# 

**Published:** 1862-03

**Authors:** 


### CHICAGO MEDICAL JOURNAL.

**Terms, $2.00 per Annum.**

### CONTENTS OF THE MARCH NUMBER.

ORIGINAL COMMUNICATIONS.

PAGE.
A Case of Amputation of the Leg Successfully Treated without Ligating the Arteries. By. H. Ristine, M. D., Marion, Linn Co., Iowa...... 325
Management of the Insane. By Geo. C. McFarland, a Student of Rush Medical College..... ........................................ 128
A Case of Hydrocele Cured by the Injection of Tinct. of Iodine. By J. N. Green, M. D., of Stilesville, Indiana.......................... 131
Case of Poisoning. By A. K. Van Horn, M. D., of Baileyville, Ill....... 132
Questions, as to the Velocity of the Blood's Movements................ 135

SELECTED.

Serous Apoplexy—Closure of Os Uteri, &c. Cases Reported to Buffalo Medical Association.......................................... 136
Variations of Animal Heat as a Cause of Disease—" Taking Cold "—Artificial Warmth. From Dr. Ware's Lectures on General Therapeutics 143
Nature and Art in the Cure of Disease.............................. 149
Researches on the Development of Tuberculous Matter. By Dr. Lavarau, Professor in Val-de-Grace.................. ................ 151

EDITORIAL.

Editorial and Miscellaneous........................................... 161

